# Prevalence of virulence genes of biofilm producing strains of *Staphylococcus epidermidis* isolated from clinical samples in Iran

**DOI:** 10.1186/s13568-015-0134-3

**Published:** 2015-08-09

**Authors:** Seyed Mostafa Solati, Elahe Tajbakhsh, Faham Khamesipour, Harish C Gugnani

**Affiliations:** Graduated of Microbiology, Shahrekord Branch, Islamic Azad University, Shahrekord, Iran; Department of Microbiology, Faculty of Basic Sciences, Shahrekord Branch, Islamic Azad University, Shahrekord, Iran; Young Researchers and Elite Club, Shahrekord Branch, Islamic Azad University, Shahrekord, Iran; Department of Microbiology and Epidemiology, Saint James School of Medicine, Bonaire, Dutch Caribbean, West Indies

**Keywords:** *Staphylococcus epidermidis*, Clinical isolates, Biofilm, Virulence genes, Microtiter assay plate

## Abstract

Coagulase negative staphylococci are recognized as opportunistic pathogens and are widespread in the environment. It is possible to prevent and control infections due to these bacteria if their virulence factors are recognized. Eighty isolates of *Staphylococcusepidermidis* (*S. epidermidis*) including 42 from urine (52.5%), 23 from blood (28.75%), 15 from dialysis bags (18.75%) were studied for biofilm production on Congo red agar (CRA). The virulence genes in *S*. *aureus* were investigated using polymerase chain reaction (PCR) with primers. Out of 80 isolates studied, 40 isolated (50%) formed black colonies (biofilm-forming strains) on CRA. In 22 of these isolates (25%) reaction was strongly positive; in 12 isolates (15%) reaction was moderately positive, and in the remaining 6 isolates, reaction was weakly positive. In the 22 isolates that had strong positive reaction and produced black colonies on biofilm, all virulent genes (*icaC, icaD, icaA icaB, icaR*) were expressed. In the 12 isolates that had moderate positive reaction, 8 expressed all genes (*icaC, icaD, icaA icaB, icaR*) expressed while the remaining 4 expressed only *ica A*, and *ica D* genes. Of the 6 isolated which had weak positive reaction, only 1 isolate (2.5%) expressed all the genes, in the other 5 isolates no gene was observed. Urinary isolates more frequently form biofilms than the isolates from other clinical samples. Statistical analysis using Chi square test showed that there was a significant correlation between the type of sample and the biofilm production (P < 0.05). The results of biofilm production on CRA were largely in agreement with microtiter plate assay and PCR assay. The capacity of bacteria to produce biofilm is an important factor in infectivity and happens via expression of *ica* genes. Recognition of bacteria that produce biofilm is thus important to control infection due to these bacteria.

## Introduction

Staphylococci are Gram positive non-motile, non-spore forming, facultative anaerobes, occurring as cocci in clusters, and are classified in two main groups, coagulase-positive and coagulase-negative (Oto 2009; Asadollahi Dehkordi et al. 2015). Coagulase negative staphylococci (CNS) are normal inhabitants of human skin and mucosa. Though frequently isolated from clinical specimens; they are often considered as non-pathogens (Oto 2009). However, CNS are being increasingly recognized in causing nosocomial and community infections. There are 40 recognized species of CNS (Rogers et al. 2009). In contrast to *Staphylococcus aureus*, virulence properties associated with *Staphylococcus epidermidis* are few and biofilm formation on the surface of materials is the most important virulence factor as demonstrated by animal model of animal infection (Fev and Olson 2010). Production of poly-*N*-acetylglucosamine (PNAG) is crucial for *S*. *epidermidis* biofilm formation and is synthesized by the gene products of the ica ADBC gene cluster. Biofilm formation protects these bacteria against the antibacterial drugs and the immune system defenses (Fev and Olson 2010). Currently *S. epidermidis* is the predominant cause of nosocomial infections because of its potential ability in biofilm formation and colonization in different surfaces (Uckay et al. 2009; Fev and Olson 2010). *Staphylococcusepidermidis* has emerged as a major nosocomial pathogen associated with infections of implanted medical devices. In the past few decades, the clinical importance, and the emergence of methicillin-resistant *S*. *epidermidis* strains have created many challenges in the treatment process (Namvar et al. 2014). Several studies have been performed on detecting virulent genes in isolates of *Staphylococcus aureus* and *S*. *epidermidis* (Gad et al. 2009; Kumar et al. 2009; Bien et al. 2011; Gomes et al. 2011). An extracellular polysaccharide adhesin represents a key virulence determinant in *S. epidermidis* and is required for biofilm formation. Production of this adhesin is encoded by the *ica* operon (De Silva et al. 2002). A recent study from Canada concerned virulence gene expression by *S*. *epidermidis* biofilm cells exposed to antibiotics (Gomes et al. 2011). The present investigation aims at detecting virulent genes in clinical isolates of *S*. *epidermidis* recovered from patients in Iran.

## Materials and methods

### Sample

Eighty isolates of *S.epidermidis* that had been referred to the medical laboratory of Kashani Hospital, Imam Ali Hospital and Hajar Hospital in Shahrekord, Iran, including 42 from urine (52.5%) from cases of urinary tract infection, 23 from blood (28.75%) from patients of septicemia, and 15 from dialysis bags (18.75%) from kidney failure patients undergoing peritoneal dialysis. Biofilm production studied by phenotypic characterization, and microtiter plate assay. Virulence genre for biofilm formation were investigated by PCR.

### Phenotypic characterization

The method employed was that described by Freeman et al. (1989). The Congo red agar medium comprised BHI (37 g/L), sucrose (50 g/L), No. 1 agar (10 g/L) and Congo Red stain (0.8 g/L). Plates of the medium were inoculated and incubated in aerobic environment for 24 h at 37°C. Under such condition, biofilm producers form black crusty colonies on CRA, whereas non-producers form red colonies.

### Microtiter Plate Assay for detection of biofilm

Biofilm production was detected using microtiter plate assay, following the procedure described by O’ Toole (O’ Toole 2011). The isolates of *S*. *epidermidis* were inoculated in 10 mL of tryptic soy broth with 0.25% glucose and incubated overnight with shaking at 37°C. Next, the cultures were diluted 1:100, and 200 µL of the diluted cultures, per well, were inoculated into 96-well polystyrene microtiter plates. After 48 h incubation at 37°C under aerobic conditions, the plates were washed three times with 300 µL distilled water. Subsequently, the plates were stained with 200 µL of 1% crystal violet, per well, for 10 min. Excess crystal violet was removed by gently washing the plate twice with distilled water. Finally, a volume of 250 µL of 95% ethanol solution, per well, was added to the plate and the optical density was measured at 570 nm. The absorbance of destaining solution was measured at 570 nm in an Elisa reader (Stat fax-2100). A well with sterile TSB or LB served as controls, whereby their ODs were subtracted from that of the experimental strains. The mean OD 570 nm value was determined using four replicates, and was considered to be adherence positive at OD 570 nm greater than or equal to 0.300 high biofilm formation, between 0.200 and 0.299, and adherence negative at OD 570 nm less than 0.100.

### Investigation of virulence genes

The virulence genes in *S*. *aureus* were investigated by PCR. The primer sequence used, the annealing temperature and the PCR program employed are given in Table 1. Purification of DNA was achieved using a Genomic DNA purification kit (Fermentas, GmbH, St. Leon-Rot, Germany) according to the manufacturer’s instruction. The total DNA was measured at 260 nm optical density according to the method described by Sambrook and Russell (2001). The PCR reactions were performed using Accupower PCR PreMix kit (BioNEER), following essentially the procedure described by Arciola et al. (2005). The PCR mix contained 20 μL of PCR PreMix. Accupower PCR PreMix component of 1U Taq DNA Polymerase, 250 Μm Each dNTP (dATP, dCTP, dGTP, dTTp), 10 mM Tris–HCl (pH = 9), 30 mM KCl and 1.5 mM MgCl2. In each reaction add 5–50 ng Templet DNA and 5–10 pmol primer. The PCR reaction for detection of 16srRNA, *icaA, ica B, ica R* and *Ica C, D* genes were performed using 10 pmol of each primer and 50 ng DNA of reaction mix. The PCR was performed using a DNA thermal cycler (Master Cycler Gradiant, Eppendrof, Germany). The amplicons were stained with ethidium bromide and electrophoresed in 1.5% agarose gel at 80 V for 30 min. PCR products were visualized and photographed using UVIdoc gel documentation systems (Uvitec, UK). The PCR products were compared against a 100 bp DNA marker (Fermentas, Germany). *Staphylococcus epidermidis* PTCC 1435 was used as a positive control.

**Table 1:** Primers used genes in *Staphylococcus epidermidis*

Gene	Primer Sequence (5′–3′)	Annealing temperature	Size of product (bp)
16s rRNA	F: CCTATAAGACTGGGATAACTTCGGG R: CTTTGAGTTTCAACCTTGCGGTCG	58	791
*Ica A*	F: ACAGTCGCTACGAAAAGAAA R: GGAAATGCCATAATGACAAC	56	103
*ica B*	F: CTGATCAAGAATTTAAATCACAAA R: AAAGTCCCATAAGCCTGTTT	56	302
*Ica C*	F: TAACTTTAGGCGCATATGTTTT R: TTCCAGTTAGGCTGGTATTG	56	400
*ica D*	Ica D F: ATGGTCAAGCCCAGACAGAG Ica D R: CGTGTTTTCAACATTTAATGCAA	56	198
*ica R*	F: TAATCCCGAATTTTTGTGAA R: AACGCAATAACCTTATTTTCC	56	469

### Statistical analysis

The data on production of biofilms by the strains of *S*. *epidermidis* was analyzed by the statistical software SPSS^®^ version 19.0 (SPSS Inc., USA). P values were calculated using the Chi square test. P < 0.05 was considered to be statistically significant.

## Results

**Fig. 1:**
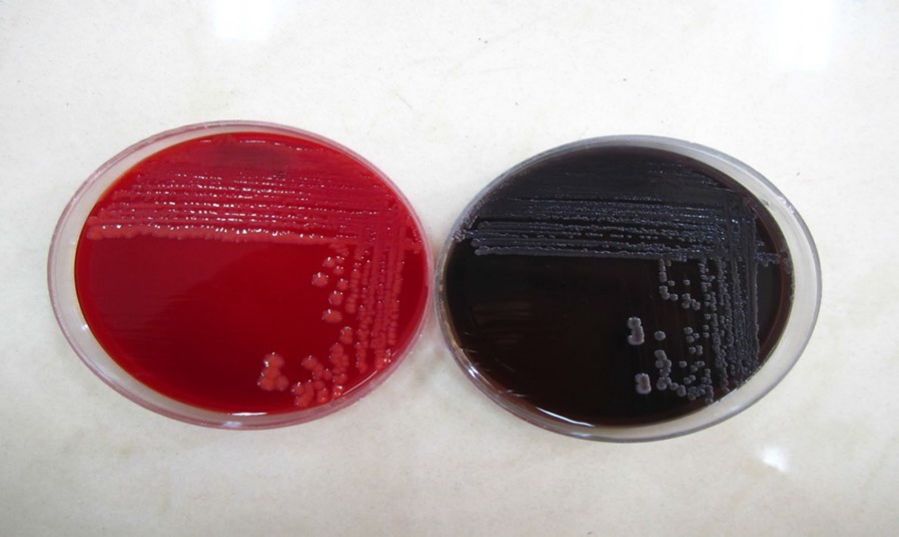
*Right* *black colonies* in a strong biofilm-producing *Staphylococcus epidermidis* isolate in the Congo red agar (CRA) medium. *Left* *red colonies* of *Staphylococcus epidermidis* isolate with no biofilm.

**Fig. 2:**
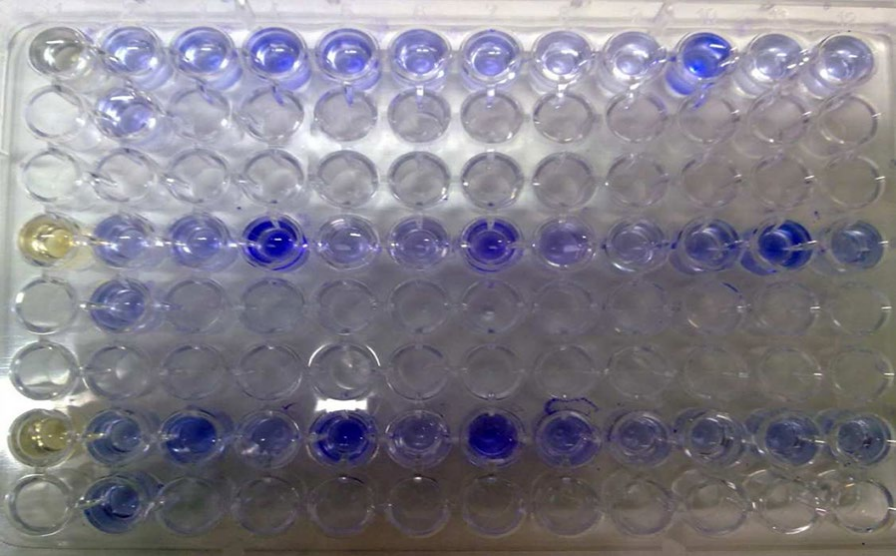
Reaction of biofilm formation in microtiter plate assay by a clinical isolate 4 of *Staphylococcus epidermidis.*

**Fig. 3:**
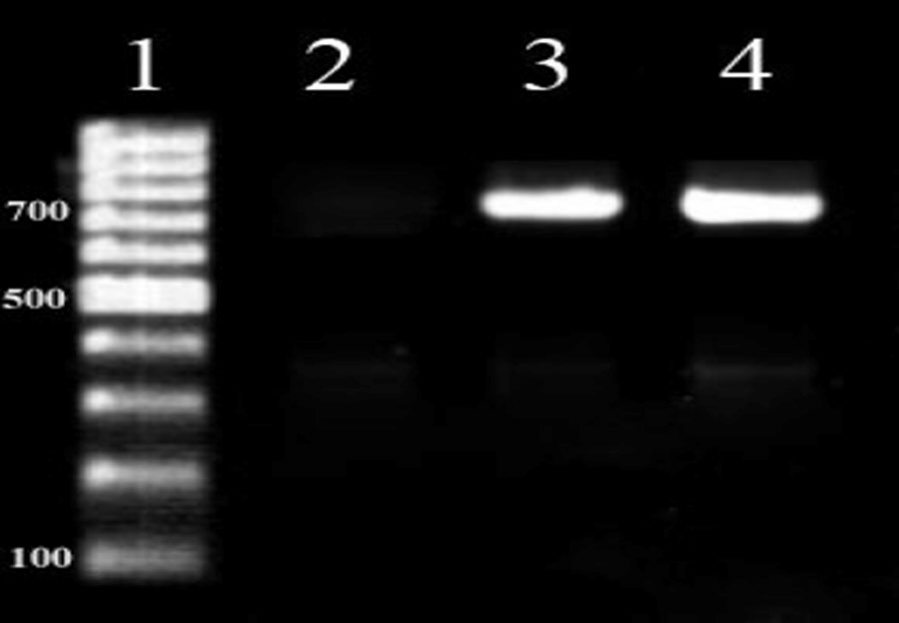
PCR Assay for Identification of 16S rRNA *Staphylococcus epidermidis.* *Number 1* DNA size ladder 100 bp (Fermentas), *number 2* negative control; *number 3* positive control (*Staphylococcus epidermidis* PTCC 1435); *number 4* positive samples.

**Fig. 4:**
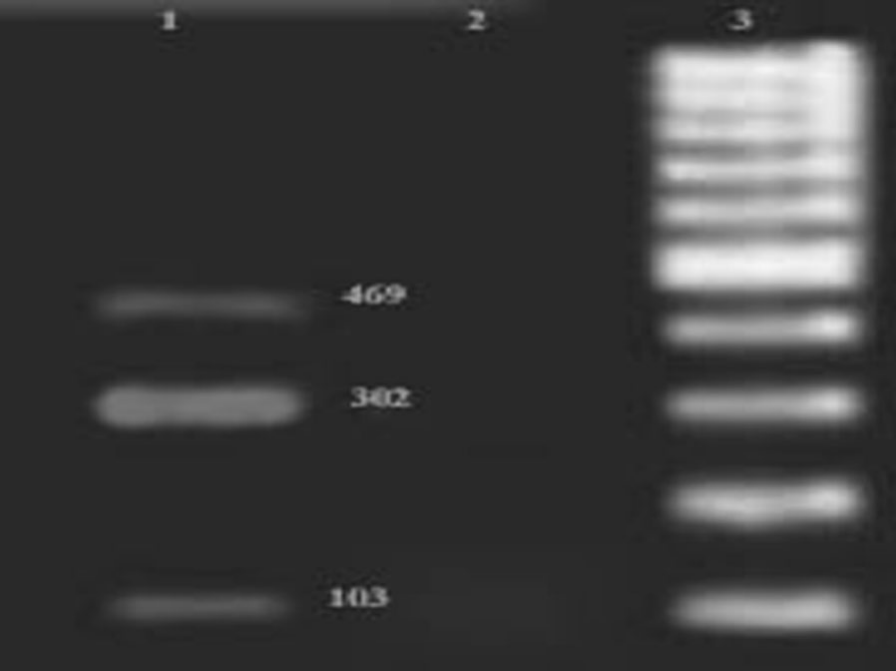
PCR Assay for Identification of icaR, icaB, icaA genes of *S. epidermidis.* *Number 1* positive samples; *number 2* negative control; *number 3* DNA size ladder 100 bp (Fermentas).

**Fig. 5:**
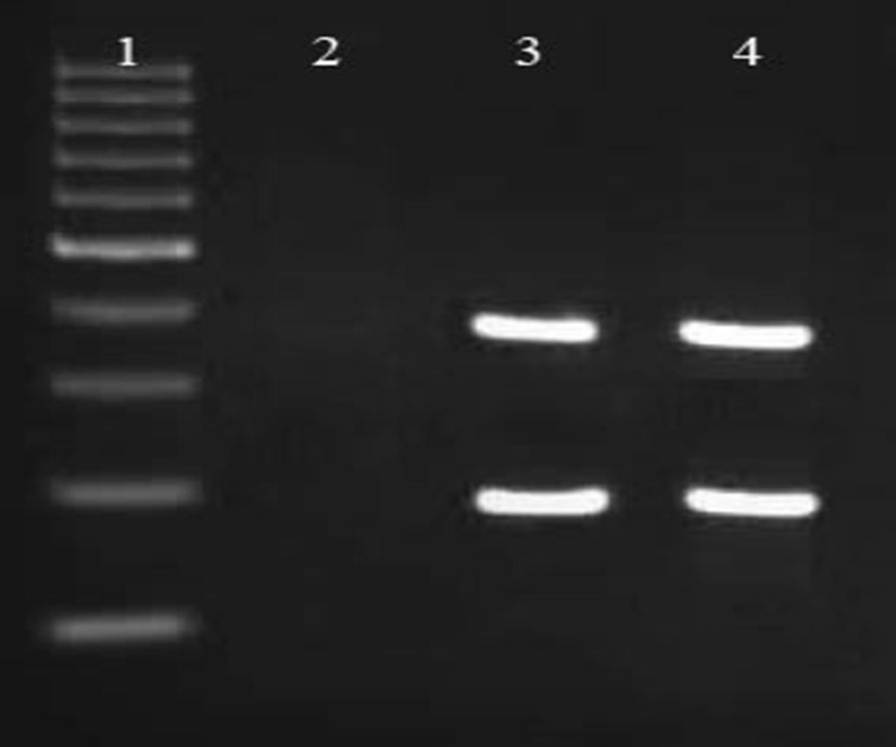
PCR Assay for Identification of icaD, icaC genes of *S. epidermidis.* *Number 1* DNA size ladder 100 bp (Fermentas); *number 2* negative control; *number 3* positive samples; *number 4* positive control (*Staphylococcus epidermidis* PTCC 1435).

Out of 80 isolates of *S*. *epidermidis* examined, 40 (50%) produced biofilms as evidenced by formation of black colonies on CRA plates (Fig. 1). The biofilm production was strong in 22 (55%) isolates, moderate in 12 (30%) and weak in 6 (15%) isolates as judged by the intensity of black colonies. The distribution of biofilm production according to the source of isolates of isolates is shown in Table 2. The positive reaction indicating biofilm formation in microtiter plate assay is shown in Fig. 2. The results of the ELISA readings on 80 isolates of *S. epidermidis* for biofilms production is shown in Table 3. The results of microtiter plate assay for biofilm production in the isolates were largely in agreement with that on CRA plates. In PCR assay all the 22 isolates produced strong reaction on CRA and in microtiter plate assay, and in 12 isolates that exhibited moderate reaction on CRA and in microtiter plate assay all the genes (*icaC, icaD, icaA icaB, icaR*). Of the 12 isolates that had moderate positive reaction on CBA and in microtiter plate assay, 8 expressed all these genes, while 4 expressed only *ica A*, and *ica D* genes, Of the 6 isolates which had weak positive reactions, only 1 isolate (2.5%) exhibited the genes while in the remaining 5 isolates no gene was observed. The detection of different genes by PCR is shown in Figs. 3, 4 and 5. Statistical analysis using Chi square test showed that a significantly higher percentage of urinary isolates formed biofilms than the isolates from other clinical samples (P < 0.05).

**Table 2:** Distribution of 40 biofilm forming strains of *Staphylococcus epidermidis* according to clinical samples

Clinical sample	No. of strains according to degree of biofilm formation						P value
	High		Moderate		Weak		
	No	%	No	%	No	%	
Urine	16	40	8	20	5	12.5	<0.05
Blood culture	2	5	1	2.5	0	0	<0.05
Dialysis catheter	4	10	3	7.5	1	2.5	<0.05
Total	22	55	12	30	6	15	<0.05

**Table 3:** The results of the ELISA readings on 80 isolates of *Staphylococcus epidermidis* for biofilms production

Number of samples	Result	Number of samples	Result	Number of samples	Result	Number of samples	Result
1	0.001	21	0.007	41	0.125	61	0.311
2	0.254	22	0.004	42	0.893	62	0.050
3	0.028	23	0.192	43	0.054	62	1.314
4	0.001	24	0.183	44	0.463	64	0.043
5	0.405	25	0.279	45	0.054	65	0.063
6	0.208	26	0.045	46	0.073	66	1.000
7	0.005	27	0.006	47	0.154	67	0.006
9	0.019	29	0.221	49	0.090	69	0.307
10	0.069	30	0.004	50	0.051	70	0.012
11	0.065	31	0.251	51	0.313	71	0.025
12	0.079	32	0.081	52	0.096	72	0.411
13	0.355	33	0.225	53	0.073	73	0.317
14	0.314	34	0.141	54	0.030	74	0.011
15	0.410	35	0.069	55	0.241	75	0.010
16	0.403	36	0.081	56	0.157	76	0.052
17	0.301	37	0.070	57	0.023	77	0.310
18	0.062	38	0.088	58	0.367	78	0.228
19	0.356	39	0.283	59	1.107	79	0.309
20	0.225	40	0.209	60	0.040	80	0.229

## Discussion

The capacity of *S*. *epidermidis* to produce biofilm is an important factor in infectivity and happens via expression of *ica* genes. The present study is the first of its kind from the Gulf region dealing with biofilm forming genes expression in clinical isolates of *S*. *epidermidis* from Iran. An earlier study from Canada concerned virulence gene expression by biofilm producing strains of *S*. *epidermidis* exposed to antibiotics (Gomes et al. 2011). From the results of our study it is evident that the genes responsible for biofilm production are present to a varying degree in the clinical isolates. As can be seen in Table 1, there was a significant relationship between the type of sample and the biofilm production, as tested by Chi square test (P < 0.05). The reactions of biofilm production on Congo red agar, and in microtiter plate assay and PCR assay were largely in agreement, though no statistical analysis was done.

In a previous study, *S*. *epidermidis* isolates recovered from catheter segments showed a higher extent of biofilm production than that isolated from urine samples (Gad et al. 2009). In our study, urinary isolates demonstrated a much higher percentage of high biofilm production than that from dialysis catheter (Table 2). However, the overall percentage of biofilm producing strains is much lower in our study than that in the one from Egypt (Gad et al. 2009). Among ica genes, *icaA* and *icaD* have been reported to play a significant role in biofilm formation in *S. aureus* and *S. epidermidis* (De Silva et al. 2002). It is significant to note that both these genes were demonstrated in our biofilm producing strains of *S*. *epidermidis*. Further research is needed to contribute to the development of biomaterials and physical electrical barriers to impede bacterial colonization, and also novel strategies for therapeutic intervention.

## Abbreviations

CRA: Congo red agar
CNS: coagulase negative staphylococci
PNAG: production of poly-*N*-acetylglucosamine
*S. epidermidis*: *Staphylococcus epidermidis*
PCR: polymerase chain reaction
